# Next step towards point-of-care molecular diagnosis of female genital schistosomiasis (FGS): evaluation of an instrument-free LAMP procedure

**DOI:** 10.3389/fpara.2024.1297310

**Published:** 2024-05-13

**Authors:** Kim J. M. van Bergen, Eric A.T. Brienen, Bodo S. Randrianasolo, Charles E. Ramarokoto, Peter Leutscher, Eyrun F. Kjetland, Angela van Diepen, Floris Dekker, Vittorio Saggiomo, Aldrik H. Velders, Lisette van Lieshout

**Affiliations:** ^1^ Department of Parasitology, Leiden University Medical Center, Leiden, Netherlands; ^2^ Department of Epidemiology, Association K’OLO VANONA, Antananarivo, Madagascar; ^3^ Department of Infectious Diseases, Aarhus University Hospital, Aarhus, Denmark; ^4^ Norwegian Centre for Imported and Tropical Diseases, Department of Infectious Diseases Discipline of Public Health, College of Health Sciences, University of KwaZulu-Natal, Durban, South Africa; ^5^ Department of BioNanoTechnology, Wageningen University, Wageningen, Netherlands

**Keywords:** *Schistosoma haematobium*, loop-mediated amplification, point-of-care molecular diagnosis, instrument-free isothermal amplification device, female genital schistosomiasis, real-time PCR

## Abstract

Detection of *Schistosoma* spp. DNA in gynaecological samples by quantitative real-time polymerase chain reaction (qPCR) is considered to be the reference diagnostic test for female genital schistosomiasis (FGS). However, qPCR needs expensive laboratory procedures and highly trained technicians. Loop-mediated amplification (LAMP) is a more field-friendly isothermal procedure for the detection of parasite-specific DNA, but it still requires electrically powered equipment. Here, we validated a *Schistosoma haematobium*-specific Sh-LAMP procedure and tested a fully instrument-free isothermal amplification using a novel low-cost, and reusable Temperature-cup (T-cup) device. Specific primers were selected based on published assays, targeting the ribosomal intergenic spacer (IGS) region of *S. haematobium*. Technical validation of the IGS-Sh-LAMP was performed using 20 negative controls, including DNA extracts of soil-transmitted helminths and *S. mansoni*, and a 10-fold dilution series (10^0^–10^−3^) of DNA extracted from a single *S. haematobium* egg (n=4). For clinical validation, the IGS-Sh-LAMP was tested on 125 DNA samples extracted from vaginal swabs of a previous FGS study in Madagascar. Results were compared with the quantification cycle value (Cq) of the standard ITS-2 targeting qPCR. Single *S. haematobium* egg DNA up to a 10^–2^ dilution and an ITS-2 Cq <35 tested positive in the IGS-Sh-LAMP. The specificity was found to be excellent (100%). In the clinical samples, IGS-Sh-LAMP showed comparable results with the qPCR, with 35.2% and 33.6% positives, respectively, and a concordance of 79.2% (99/125). Of the 12 false-negatives, 5 corresponded to the 7 qPCR positive samples with very low DNA levels (Cq ≥35). On the other hand, IGS-Sh-LAMP detected 14 additional cases that were not detected by qPCR. The T-cup IGS-Sh-LAMP performance was evaluated in a representative sub-selection (n=10) of IGS-Sh-LAMP positive clinical samples. The T-cup IGS-Sh-LAMP was found to be a very user-friendly method, but in different runs, it missed 1 to 4 of the 10 IGS-Sh-LAMP positive samples, specifically those with a low DNA load. Our results show that the IGS-Sh-LAMP is a suitable alternative to the ITS-2 qPCR for the diagnosis of FGS in gynaecological samples, with high potential for the T-cup as a fully instrument-free isothermal amplification device for point-of-care diagnosis in low-resource settings.

## Introduction

Schistosomiasis is a neglected tropical disease (NTD) caused by parasitic trematodes of the genus *Schistosoma* (*S.*), which afflicts around 250 million people within 78 countries (Colley et al., 2014; World Health Organization, 2022). The majority of the population at risk lives in rural areas of sub-Saharan Africa, where *S. haematobium* is the predominant species (Hotez and Kamath, 2009; Brindley and Hotez, 2013) and cause of urogenital schistosomiasis. Urogenital pathologies occur as a result of eggs produced by the female worms that are trapped in the venous plexus of the bladder and/or genital organs. Female genital schistosomiasis (FGS) refers to characteristic clinical manifestations, which are caused by an inflammatory reaction to *S. haematobium* eggs trapped in the female genital tract. Early symptoms of the disease are vaginal discharge, genital itching, contact bleeding, and abdominal pain. When left untreated, symptoms can evolve towards ulceration or swelling of the vulva and vagina, infertility, spontaneous abortion, and damage to the reproductive organ. In addition, FGS has also been associated with cervical dysplasia, sexually transmitted infections, and HIV transmission (Leutscher et al., 2008; Engels et al., 2020; Sturt et al., 2020a; Rafferty et al., 2021; Bustinduy et al., 2022).

To successfully treat, control, and eventually move towards the elimination of FGS, accurate, specific, and sensitive diagnostic tests with usage in low-resource settings are crucial. The ideal diagnostic test should be performed at the point-of-care, so that treatment can be started without delay, and should not depend on laboratory services or highly trained staff as patients in rural areas may have difficulties accessing clinics where laboratory services are available (Peeling and Mabey, 2010; Mashamba-Thompson et al., 2018). Currently, there is no reference (gold) standard for the laboratory diagnosis of FGS, except identification of *S. haematobium* eggs in a biopsy taken from a genital lesion. However, biopsy-based methods not only need specialised training and instruments, the procedure is also highly invasive and therefore not preferred, specifically not in communities with high HIV infection risk (Poggensee et al., 2001; Kjetland et al., 2012; Bustinduy et al., 2022). Alternatively, Nucleic Acid Amplification Tests (NAATs) such as real-time polymerase chain reaction (qPCR) have been shown to be highly specific and sensitive in the detection of *Schistosoma* spp. DNA within samples such as vaginal lavages or swabs acquired from the genital tract (Kjetland et al., 2009; Pillay et al., 2020; Sturt et al., 2020b; Bustinduy et al., 2022; Ursini et al., 2023). Despite its diagnostic potential, qPCR is not often used for the diagnosis of FGS in low-resource settings as this approach requires a well-equipped laboratory with skilled staff (Yager et al., 2008; Pai et al., 2012).

Isothermal DNA amplification methods, such as loop-mediated isothermal amplification (LAMP) and Recombinase Polymerase Amplification (RPA) are attractive alternatives to PCR-based methods, as they are rapid, relatively user-friendly, cost-effective, and need only minimal equipment, making them better suited for low-resource setting (Moehling et al., 2021). Several studies have already shown the potential of LAMP and RPA as useful tools for the rapid detection of pathogenic agents of infectious diseases, such as malaria, leishmaniasis, tuberculosis (Iwamoto et al., 2003; Poon et al., 2006; Takagi et al., 2009), and schistosomiasis (Wang et al., 2011; Hamburger et al., 2013; García-Bernalt Diego et al., 2021a; García-Bernalt Diego et al., 2021b; Archer et al., 2022; Mesquita et al., 2022). Although isothermal methods are popular, the currently available equipment for these methods is still expensive, and in particular, for the LAMP, electrically powered equipment is essential to maintain the temperature required for amplification. Even when more simple electronic boards are used, they still need electrical power, and, at the end of their life time will contribute to e-waste (Velders et al., 2018). To further simplify molecular diagnostics procedures, Velders et al. (2022) have recently developed a novel instrument-free and affordable LAMP device “T-cup” that can perform isothermal nucleic acid amplification at 65°C. This non-electronic, low-cost, and reusable Temperature-cup (T-cup) is based on a Phase Change wax material placed in an aluminium coffee pod. When the T-cup is placed in boiling water and the heat is turned off, the Phase Change Material (PCM) will increase its temperature to 65°C and will keep it constant for 30–35 minutes. The T-cup has the potential for use in a resource-limited setting, even when there is no access to electricity and/or instrumentation.

In this study, we validated a previously described *S. haematobium*-specific Sh-LAMP procedure targeting the ribosomal intergenic spacer (IGS) region (IGS-Sh-LAMP) and evaluated the performance of the IGS-Sh-LAMP employed in combination with the T-cup system (T-cup IGS-Sh-LAMP) (Gandasegui et al., 2015; Crego-Vicente et al., 2021).

## Materials and methods

### Sources of samples and ethical approval

Fresh *Schistosoma* eggs were received from the *S. mansoni* and *S. haematobium* life cycles, maintained in hamsters at the Department of Parasitology at the LUMC in accordance with the project license that the Dutch Central Authority approved for Scientific Procedures on Animals (CCD) (animal license number AVD11600202215904).

All human samples used were fully anonymised and samples were excluded if the patient or study participant had indicated that no material could be stored or used for any additional diagnostic research purposes, even when anonymised, as regulated by law and stated in “Human tissue and medical research: code of conduct for responsible use” (2011). Human stool samples positive for *S. mansoni* or for any of the soil-transmitted helminths *Ascaris spp*, *Necator americanus*, *Strongyloides stercoralis* or *Trichuris trichiura* were obtained via participation at the external quality assessment scheme for PCR-based detection of DNA of helminths of the Dutch Foundation for Quality Assessment in Medical Laboratories (SKML) (Cools et al., 2020). Human stool, urine, and whole blood samples negative for *Schistosoma* were kindly provided by the clinical microbiology department of the LUMC without additional clinical or patient-related information. DNA samples extracted from vaginal mucosa swabs originated from a previously published FGS study performed in an *Schistosoma*-endemic region on Madagascar, for which the ethical clearance was granted by the Committee of Ethics at the Ministry of Health in Madagascar (number 031-CE/MINSAN 4 June 2010). Further details of the Madagascar study are given at the original publication (Randrianasolo et al., 2015).

### Single *Schistosoma* egg sample preparation

*S. haematobium* and *S. mansoni* eggs were obtained by collagenase B digestion of *Schistosoma-*infected hamster gut tissue (intestine for *S. haematobium* and liver and intestines for *S. mansoni*), followed by extensive washing with PBS containing 1.7% NaCl. To obtain DNA from a single *Schistosoma* egg, a fresh suspension of 300 µL, containing approximately 2.000 eggs, was placed at the bottom of a glass Petri dish. PBS was added until the bottom of the dish was completely covered. Mature eggs were individually selected using a stereo microscope (Carl Zeiss Stemi Sv11 Stereoscope Microscope, Carl Zeiss AG, Germany) and transferred via a 10-µL single channel manual micropipette to a clean Eppendorf tube with 200 μL PBS. Each tube was checked by microscopy for the presence of a single egg. Thereafter, bead-beating was performed by adding 0.8 gram of 1.4 mm Ceramic beads (Qiagen, Hilden, Germany) followed by two rounds of 5 min bead-beating (TissueLyser LT, Qiagen) at 50 oscillations/s. Each tube was stored for a minimum of 12 h at −20°C before further processing.

### DNA extraction

DNA was extracted from each 200-µL sample as described before, using the QIAamp DNA mini kit (Qiagen, Hilden, Germany), following the manufacturer’s protocol. Before adding the Tissue Lysis buffer (ATL, Qiagen) and the proteinase K (Qiagen), each sample was heated for 10 min at 100°C (Obeng et al., 2008; Meurs et al., 2015). Phocin Herpes Virus-1 (PhHV), obtained from European Virus Archive Global, Erasmus MC Rotterdam, was added to each sample as an internal control and for the detection of potential inhibition of amplification (Obeng et al., 2008; Gandasegui et al., 2015).

### *Schistosoma* qPCR

All DNA samples were analysed by the previously published ITS-2 qPCR for the detection of *Schistosoma* DNA (Obeng et al., 2008; Meurs et al., 2015). In addition, the DNA samples used for the technical validation of the IGS-Sh-LAMP were tested for the presence of *S. haematobium*-specific DNA by the Dra1 PCR (Hamburger et al., 2001; Cnops et al., 2013) and for the presence of *S. mansoni-*specific DNA by the Sm1–7 PCR (Wichmann et al., 2009; Cnops et al., 2012).

### DNA samples used for technical and clinical validation IGS-Sh-LAMP

Twenty DNA samples were used to test the specificity of the IGS-Sh-LAMP, including 10 samples extracted from human stool, 4 from human urine, 3 from whole blood and 3 from a single *S. mansoni* egg (**Table 1**, numbers 1–20). These samples were determined negative for *S. haematobium* in the Dra1 PCR, while the 5 *S. mansoni* samples were positive in the ITS-2 and the Sm1–7 PCR. For sensitivity, DNA’s extracted from a single *S. haematobium* egg (n=4) were used, including a serial 10-fold dilution (10^–1^–10^–3^) prepared in AE-buffer (Qiagen, Hilden, Germany) (**Table 1**, numbers 21–36). These 16 DNA samples were positive in the ITS-2 qPCR and positive for *S. haematobium* in the Dra1 PCR, while negative for *S. mansoni* in the Sm1–7 PCR. The 16 samples were tested with the IGS-Sh-LAMP in two independent runs. To evaluate the reproducibility at low DNA concentrations, two DNA samples (number 32 and 36, **Table 1**) were tested six additional times with the IGS-Sh-LAMP.

**Table 1:** Characteristics of DNA templates used for technical validation of the IGS-Sh-LAMP.

	Sample number	Specimen type	Positive for:	ITS-2 qPCR Cq-value
**Specificity**	1	Stool	*S. mansoni*	20.5
	2	Stool	*S. mansoni*	22.7
	3	Stool	*Ascaris spp* *Trichuris trichiura*	Negative
	4	Stool	*Ascaris spp*	Negative
	5	Stool	*Ascaris spp* *Trichuris trichiura* *Necator americanus* *Strongyloides stercoralis*	Negative
	6	Stool	*Necator americanus* *Strongyloides stercoralis*	Negative
	7	Stool	*Necator americanus* *Strongyloides stercoralis*	Negative
	8	Stool	–	Negative
	9	Stool	–	Negative
	10	Stool	–	Negative
	11	Urine	–	Negative
	12	Urine	–	Negative
	13	Urine	–	Negative
	14	Urine	–	Negative
	15	Whole blood	–	Negative
	16	Whole blood	–	Negative
	17	Whole blood	–	Negative
	18	gDNA of single egg	*S. mansoni*	21.8
	19	gDNA of single egg	*S. mansoni*	22.0
	20	gDNA of single egg	*S. mansoni*	25.3
**Sensitivity**	21	gDNA of single egg	*S. haematobium*	26.8
	22	gDNA of single egg	*S. haematobium*	27.0
	23	gDNA of single egg	*S. haematobium*	27.0
	24	gDNA of single egg	*S. haematobium*	27.0
	25	gDNA egg dilution 10^–1^	*S. haematobium*	29.9
	26	gDNA egg dilution 10^–1^	*S. haematobium*	30.2
	27	gDNA egg dilution 10^–1^	*S. haematobium*	30.2
	28	gDNA egg dilution 10^–1^	*S. haematobium*	30.8
	29	gDNA egg dilution 10^–2^	*S. haematobium*	33.2
	30	gDNA egg dilution 10^–2^	*S. haematobium*	33.4
	31	gDNA egg dilution 10^–2^	*S. haematobium*	33.8
	32	gDNA egg dilution 10^–2^	*S. haematobium*	35.0
	33	gDNA egg dilution 10^–3^	*S. haematobium*	35.6
	34	gDNA egg dilution 10^–3^	*S. haematobium*	35.6
	35	gDNA egg dilution 10^–3^	*S. haematobium*	35.7
	36	gDNA egg dilution 10^–3^	*S. haematobium*	36.0

gDNA, genomic DNA.

For the clinical validation of the IGS-Sh-LAMP, DNA samples previously extracted from 125 vaginal mucosa swabs were used of which 33.6% (42/125) were positive by the ITS-2 qPCR, with Cq-values ranging from 20.4 to 37.1 (median 28.7) (Randrianasolo et al., 2015). From here on they are indicated as the samples of “the FGS Madagascar study”. A receiver operating characteristic (ROC) curve was made to assess the association between the specificity and sensitivity of the IGS-Sh-LAMP at different amplification time cut-off points, using the ITS-2 qPCR outcome as the reference standard.

### IGS-Sh-LAMP assay

#### IGS-Sh-LAMP primers

A previously published IGS LAMP primer set was implemented and validated for the detection of *S. haematobium* DNA, targeting 199 bp of the ribosomal IGS sequence (**Table 2**) (Gandasegui et al., 2015; Crego-Vicente et al., 2021). To confirm the specificity of the selected IGS LAMP primers for annealing exclusively with *S. haematobium*, a BLASTN search and alignment analysis using default settings was carried out in the online database of NCBI (NCBI; http://blast.ncbi.nlm.nih.gov/Blast.cgi). IGS LAMP primers were synthesised commercially (Integrated DNA Technologies/IDT, Coralville, Iowa, United States) using standard desalting and resuspended in AE-buffer (Qiagen, Hilden, Germany) to a stock concentration of 100 µM. A 10X primer mix was prepared so that adding 2.5 µL of primer mix to a LAMP reaction yielded the following final primer concentrations: 0.2 µM F3/B3, 0.4 µM LB/LF, and 1.6 µM FIP/BIP.

**Table 2:** IGS LAMP primer set for the amplification of *S. haematobium*.

Target	Primer name	Sequence (5’ to 3’)	Reference
**IGS**	FIP	5’- TACCCCTAACTTCGTGGTCTCCCCCCCTTATTTTAGGGTGC -3’	Gandasegui et al. (2015), Crego-Vicente et al. (2021)
	BIP	5’- CTCCCTATATAACATGGCGAGTAAGACTATGAAATCAGTGTTTTTCGG -3’	
	F3	5’- CTTTCTAAGCCCGCGATA -3’	
	B3	5’- GCGCATTACACTTGGTCT -3’	
	LF	5’- GGTGCGCTTTGTTTTCCGT-3’	
	LB	5’- ACCATGTGTAAAGCGCGTCAAA-3’	

FIP, forward internal primer; BIP, backwards internal primer; F3, forward external primer; B3, backwards external primer; LF, loop forward primer; LB, Loop backward primer.

#### IGS-Sh-LAMP amplification and detection

Each LAMP reaction was carried out in clear 0.2 mL strips (Bio-Rad, Hercules, CA, USA) with a final volume of 25 µL containing 12.5 µL WarmStart LAMP Kit (DNA & RNA, E1700, New England Biolabs, MA, USA), 2.5 µL primer mix (10X), 0.5 µL fluorescent dye (50X, concentration not given, E1700, New England Biolabs), 4.5 µL of nuclease-free water (Qiagen, Hilden, Germany) and 5 µL template DNA. The IGS-Sh-LAMP was performed in a CFX-96 Touch Real-Time PCR Detection System (Bio-Rad, Hercules, CA, USA) with the following settings: 61 cycles of 30 seconds at 65°C (total time: 45.1 minutes, including plate read every cycle and ramping time) with the registration of fluorescence signal each cycle in the FAM channel. After the 61 cycles, the analysis was followed by a melting curve step: gradual temperature increase from 65 to 98°C (0.5°C per 5 seconds). A positive (*S. haematobium* gDNA) and negative control (AE-buffer) were included in all runs. Cq-values were determined by the software’s Cq Determination mode (settings: Single Threshold set at 1000) and calculated to amplification time in minutes by multiplying the Cq-value with 44 seconds, which is the total time of 1 cycle plus plate read. The result of the IGS-Sh-LAMP was determined positive if the amplification time was ≤ 30 minutes and negative when there was no amplification time or the amplification time was > 30 minutes.

### Evaluation of the instrument-free T-cup Sh-LAMP (T-cup IGS-Sh-LAMP)

**Figure 1:**
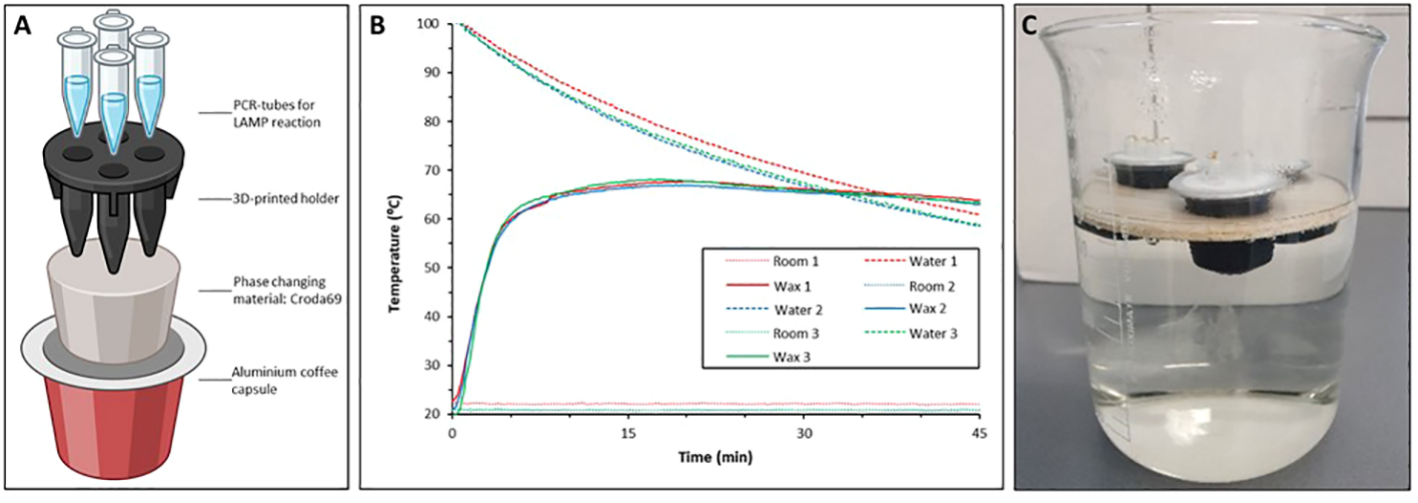
**(A)** Schematic representation of the T-Cup v 2.0 in which a 3D printed holder physically separates the vials from the wax, avoiding overheating issues; **(B)** Triplicate repetition of temperature in the PCR vials in a LAMP run. **(C)** IGS-Sh-LAMP reaction performed in a T-cup. The T-cup was placed on a floating holder and incubated in hot water for 30 minutes.

To evaluate the performance of the instrument-free T-cup IGS-Sh-LAMP, 10 IGS-Sh-LAMP positive DNA samples of the FGS study were randomly selected, representing a broad range of amplification times in the IGS-Sh-LAMP (10.4–22.2 minutes). Compared to the previously reported T-cup (Velders et al., 2022), the version used here “T-cup v2.0” has a few improvements: The PCM material used is the Croda69 (Croda Europe, Gouda, The Netherlands). This biobased resin is more environmentally friendly than the previously used wax. In addition, we 3D-printed a holder for four standard 200 µL PCR vials (Thermofisher, Bleiswijk, The Netherlands), so they are not in direct contact with the resin, decreasing the risk of local overheating and deactivation of the LAMP enzyme (**Figure 1A**, **Supplementary Material**). The procedure for manufacturing the T-cup is the same as our previous work. In short: A clean aluminium coffee capsule was filled with 6 grams of Croda69. This was heated in a water bath until all the wax was liquid. At this point, the 3D-printed holder (printed in ABS at 100% infill) was immersed in the capsule and the capsule was left at room temperature until cold. When placed back in a water bath filled with boiled water, the T-cup will maintain a constant temperature of 65°C (**Figure 1B**). To prevent contamination, 1 μL of SYBR Green I (1:10 dilution, SYBR^®^ Green I nucleic acid gel stain, Sigma Aldrich) was added to the inner side of the tube cap before amplification, as described by Lai et al. (2021). Water was heated to 100°C and 700 mL of water was transferred to a 1L glass beaker. To keep the T-cup floating in water, it was placed in a floating holder made out of balsa wood and incubated in the hot water for 30 minutes (**Figure 1C**). After 30 minutes the reaction tubes were taken out and inverted to allow mixing with the SYBR Green I. The 10 samples were analysed in two separate experiments and results were interpreted directly by the naked eye. A positive (*S. haematobium* gDNA) and negative control (AE-buffer) were included to validate run validity.

## Results

### Specificity and sensitivity of the IGS-Sh-LAMP

When testing specificity, none of the 20 *S. haematobium* negative DNA samples summarised in **Table 1** showed amplification or melting curves in the IGS-Sh-LAMP assay.

**Figure 2:**
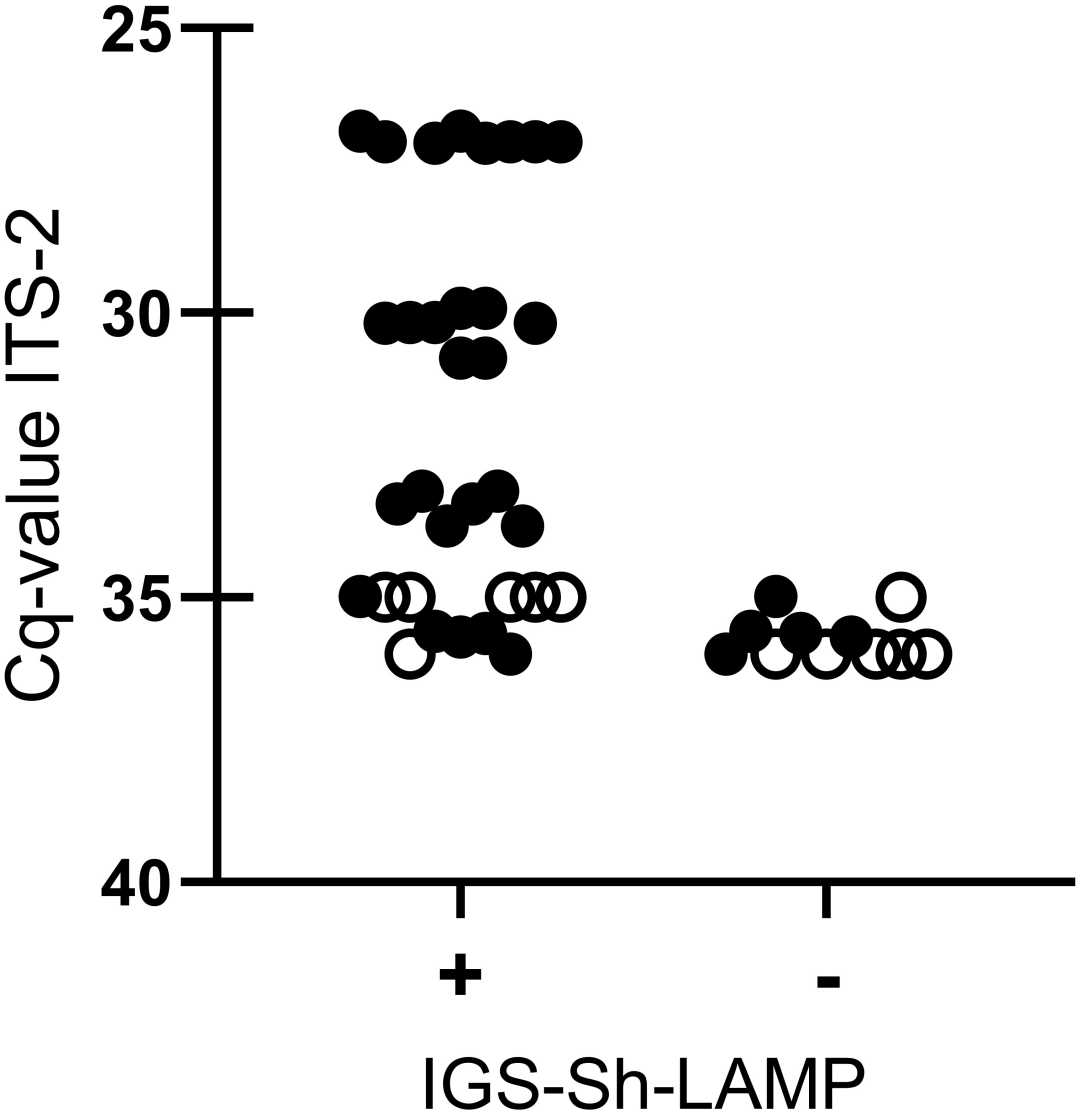
Comparison between IGS-Sh-LAMP and ITS-2 qPCR based on different dilutions (10^0^–10^–3^) of DNA extracted from a single *S. haematobium* egg (n=4). IGS-Sh-LAMP positive results and negative results are plotted on the x-axis against the Cq-value determined by the ITS-2 qPCR on the y-axis. Each LAMP measurement is represented as an individual dot. All 16 DNA samples were tested in duplicate in the IGS-Sh-LAMP (black dots), while for reproducibility testing 2 DNA samples with a low DNA loads (Cq-value 35 and Cq-value 36.) were tested 6 additional times (open dots).

When sensitivity was tested on 16 DNA samples extracted from a single *S. haematobium* egg, all DNA samples diluted up to 10^–2^ tested positive in the IGS-Sh-LAMP in both runs, with the exception of sample 32 (Cq-value 35) which tested positive in one of the two runs. The four DNA samples diluted 10^–3^ were detected in 50% by the IGS-Sh-LAMP. Sample 32 (Cq-value 35) and 36 (Cq-value 36) tested six additional times were positive in 5/6 (83.3%) and 1/6 (16.7%), respectively (**Figure 2**).

### Clinical validation of the IGS-Sh-LAMP

**Figure 3:**
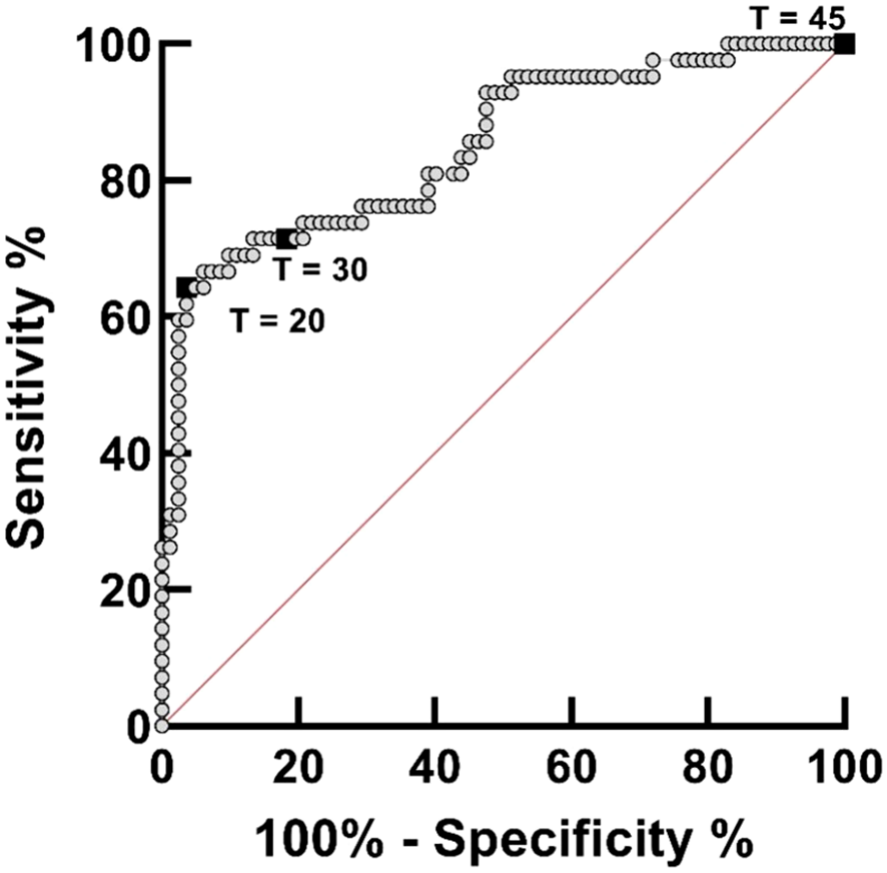
Receiver operating curve (ROC) analysis for assessing the performance of the IGS-Sh-LAMP on DNA extracted from 125 vaginal mucosa swabs originating from a previously published FGS study performed in Madagascar, using the ITS-2 qPCR as reference test. The ROC curve was created by plotting the true-positive rate (sensitivity) on the y-axis against the false-positive rate (100% – specificity) on the x-axis. The 45**°**C diagonal red line serves as a reference line of a ROC curve for a test with no discriminatory ability. The amplification time cut-off points of 20, 30, and 45 minutes are highlighted by black boxes.

*S. haematobium* DNA was detected by IGS-Sh-LAMP in 35.2% (44/125) of the DNA samples of the FGS Madagascar study (**Table 3**). Of those samples with a Cq-value <35 in the ITS-2 qPCR, 80% were positive in the IGS-Sh-LAMP, compared to 28.5% (2/7) of the samples with a Cq ≥35 in the ITS-2 qPCR. On the other hand, 16.9% (14/83) of the ITS-2 qPCR-negative samples were positive in the IGS-Sh-LAMP. The relationship between clinical sensitivity and specificity of the IGS-Sh-LAMP was further analysed using a ROC curve (**Figure 3**). An amplification time cut-off point of 30 minutes showed a sensitivity of 71.4% (CI: 56.4–82.8), and a specificity of 81.7% (CI: 71.9–88.6) compared to a sensitivity of 64.3% (CI: 49.2–77.0) and specificity of 96.3% (CI: 89.8–99.0s) for an amplification time cut-off point of 20 minutes. An amplification time cut-off point of 45 minutes showed 100% sensitivity, but only 1% specificity. The AUC of the ROC curve was determined 0.84.

**Table 3:** Percentage positive clinical FGS study samples in the IGS-Sh-LAMP (30 minutes) compared to the ITS-2 qPCR.

	Percentage of positives in the IGS-Sh-LAMP	Percentage of positives in the ITS-2 qPCR
**Total** **(n=125)**	35.2 (44/125)	33.6 (42/125)
**ITS-2 qPCR,** **Cq < 35** **(n=35)**	80.0 (28/35)	100.0 (35/35)
**ITS-2 qPCR,** **Cq ≥ 35** **(n=7)**	28.5 (2/7)	100.0 (7/7)
**ITS-2 qPCR, Negative (n=83)**	16.9 (14/83)	0.0 (0/83)

### Instrument-free IGS-Sh-LAMP (T-cup IGS-Sh-LAMP)

**Figure 4:**
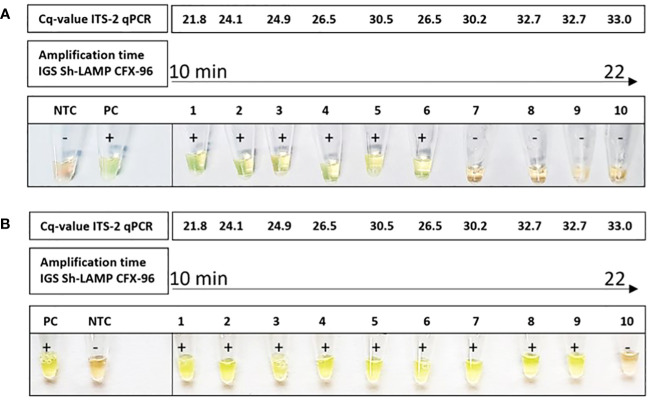
Amplification of *S. haematobium* DNA using the T-cup LAMP system in combination with SYBR green I in 10 IGS-Sh-LAMP positive samples of the FGS Madagascar study. The DNA samples are ranked from lowest amplification time (10 minutes) to the highest amplification time (22 minutes) measured using the IGS-Sh-LAMP in the CFX-96. The ITS-2 Cq-values of the ten samples are indicated at the top. A light-green color indicates the presence of *S. haematobium* DNA and the orange color indicates the absence of *S. haematobium* DNA. *S. haematobium* was detected in 6/10 **(A)** and 9/10 **(B)** samples using the T-cup system.

The T-cup LAMP system was tested on 10 randomly selected positive DNA samples from the FGS Madagascar study in two separate experiments, showing a positive signal in 6/10 and 9/10 samples, respectively (**Figure 4**). By performing the IGS-Sh-LAMP on the CFX-96, amplification times of the 10 samples were calculated based on Cq-values and compared with the colorimetric results when using the T-cup LAMP system. In both T-cup LAMP experiments, the sample(s) with the highest Cq-value in the reference ITS-2 qPCR test were not detected within 30 minutes, but were positive in the IGS-Sh-LAMP performed on the CFX-96. These results indicate a lower sensitivity for the IGS-Sh-LAMP when performed in the T-cup compared to the IGS-Sh-LAMP performed in the CFX-96.

## Discussion

A major challenge in treatment and control of FGS is the development and availability of specific, sensitive, and affordable diagnostic tools for the point-of-care detection of *S. haematobium* in resource-limited settings. In this study, we validated a *S. haematobium*-specific Sh-LAMP targeting the ribosomal intergenic spacer region and tested a fully instrument-free isothermal amplification by using a novel low-cost and reusable T-cup device.

The IGS-Sh-LAMP showed 100% specificity with no cross-reactivity against *S. mansoni* or any of the other included soil-transmitted helminth targets. This finding is of medical importance as coinfections often occur in *S. haematobium* endemic areas resulting in co-existence of different parasites in field samples (Opara et al., 2021). These results align with the study of Gandasegui et al. (2015) in which a panel of different parasites including helminths and protozoa was examined. The sensitivity of the IGS-Sh-LAMP was based on Cq-values determined by the previously published ITS-2 qPCR for the detection of *Schistosoma* DNA in clinical samples. In terms of Cq-value, the IGS-Sh-LAMP was found to be less sensitive than the ITS-2 qPCR, specifically in samples with very low DNA loads (Cq-values 35–36). However, it should be noted that a single *S. haematobium* egg (ITS-2 qPCR Cq-value 27) could be detected within 11 min with the IGS-Sh-LAMP, and a single *S. haematobium* egg DNA up to a 10^–2^ dilution (ITS-2 Cq- values <35) was detected in all performed tests. *S. haematobium* egg DNA diluted 10^–3^ with Cq-values of 35–36, so correlating with very low DNA loads was detected in only half of the performed tests. Furthermore, when assessing the reproducibility of two samples with very low DNA loads (Cq-values 35 and 36), *S. haematobium* DNA was detected in 83.3% (5/6) and 16.7% (1/6), respectively (**Figure 2**).

For clinical validation, the IGS-Sh-LAMP was performed on 125 DNA samples extracted from vaginal swabs of a previous FGS study in Madagascar and compared with the results of the ITS-2 qPCR (Randrianasolo et al., 2015). The IGS-Sh-LAMP showed comparable results with the ITS-2 qPCR, with 35.2% and 33.6% positives, respectively, and a concordance of 79.2% (99/125) (**Table 3**). It is worth noting that these clinical samples reflected a broad range in infection intensity, with ITS-2 Cq-values ranging from 20.4 to 37.1 (median 28.7), so including samples with a DNA quantity approximately 10–100 times greater than that of a single egg, while still yielding a specific LAMP signal. Because of the type of material, e.g. vaginal lavage, the number of actual eggs present in the local tissue could not be determined.

Most of the false-negative results with the IGS-Sh-LAMP could be correlated back to low DNA loads, as these samples showed ITS-2 Cq-values ≥ 35. In addition, other false-negatives may be related to the presence of inhibitors that might have been introduced in clinical samples during sample processing and/or DNA extraction thereby influencing the efficacy of the LAMP-reaction (Francois et al., 2011; Wang, 2020). However, it should be noted that the inclusion of the *Bst* 2.0 polymerase in our LAMP-mix provides enhanced inhibitor tolerance (Nam et al., 2023). Nonetheless, Nwe et al. (2024) reported LAMP inhibition attributed to various inhibitors, despite the use of the *Bst* 2.0 enzyme. On the contrary, the IGS-Sh-LAMP detected 14 additional cases, which may potentially be positive cases missed by the ITS-2 qPCR (**Table 3**). The possibility of false positives by the IGS-Sh-LAMP cannot be excluded. Nevertheless, the specificity of the IGS-Sh-LAMP assay was tested against a broad range of negative controls, including *S. mansoni* and other soil-transmitted helminth targets. Additionally, the IGS-Sh-LAMP was performed in a controlled laboratory setting, which limits the likelihood of cross-contamination.

There are a few limitations of this study that still need to be addressed. The main focus of the study was pointed towards the amplification and detection of *Schistosoma* DNA by the IGS-Sh-LAMP assay. However, to implement a molecular test at the point-of-care level in a low-resource setting, a rapid and field-appropriate DNA extraction method is essential. Field-friendly methods such as the SwiftX DNA extraction have already shown potential for application in low-resource settings due to their speed, simplicity, low resource needs and high input volume (Rostron et al., 2019). Therefore, the SwiftX would be an attractive method in combination with the IGS-Sh-LAMP, especially for the detection of *S. haematobium* eggs in large volume urine samples. Similarly, Gandasegui et al. (2015) proposed the low-cost Rapid-Heat LAMPellet method, employing heated urinary pellets for the detection of *S. haematobium* in clinical urine samples with LAMP. Another limitation is the requirement for cold and long-term storage of IGS-Sh-LAMP reagents, often infeasible in low-resource setting. To overcome this limitation, lyophilisation of IGS-Sh-LAMP reagents should be validated to provide greater accessibility of the IGS-Sh-LAMP to the field (García-Bernalt Diego et al., 2019; Song et al., 2022). It should also be mentioned that the IGS-Sh-LAMP is a rapid and simple alternative to qPCR, but costs still might be a limiting factor. Based on our own calculations we estimate the consumable costs of the IGS-Sh-LAMP to be 2.50 euro per sample, which is similar to that of the ITS-2 qPCR, which we estimate to be 2.40 euro per sample. These calculations do not include the costs of DNA extraction or a PCR device, meaning that further cost reduction remains one of the major goals before NAATs will be widely available for the diagnosis of schistosomiasis.

A first step in reducing extra costs and applying the IGS-Sh-LAMP in low-resource settings is performing the IGS-Sh-LAMP in the T-cup system. The T-cup IGS-Sh-LAMP provides a user-friendly and instrument-free procedure with high potential for use in low-resource settings, as results can be observed by the naked eye and detection is accomplished within a closed system avoiding cross-contamination. However, the T-cup IGS-Sh-LAMP showed a lower sensitivity compared to the IGS-Sh-LAMP performed in the CFX-96, missing 4/10 and 1/10 IGS-Sh-LAMP positive samples in two independent runs (**Figure 4**). These samples showed the highest amplification times and Cq-values in the ITS-2 qPCR, indicating that the consistency of the T-cup is not yet optimal for samples with low DNA loads. Still, these results suggest that the T-cup based IGS-Sh-LAMP would be an appropriate procedure for the point-of-care detection of moderate to high *S. haematobium* infection intensities, as a single *S. haematobium* egg (ITS-2 qPCR Cq-value of 27) can easily be detected with the IGS-Sh-LAMP in the T-cup system (**Figure 4**). The T-cup IGS-Sh-LAMP could for example be applied for the detection of *S. haematobium* egg DNA in the sediment of collected large urine volumes. To fully assess its point-of-care diagnostic potential, the T-cup IGS-Sh-LAMP should be further and more elaborately evaluated in schistosomiasis-endemic settings.

An approach to further reduce costs could involve utilising a different fluorescent dye for visualisation of the LAMP products. Avila et al. (2021) demonstrated cost reduction by employing malachite green as an alternative for SYBR Green I.

In conclusion, our study showed the IGS-Sh-LAMP to be a suitable qualitative alternative for the ITS-2 qPCR for the diagnosis of FGS in gynaecological samples. Furthermore, our results indicate that the T-cup system in combination with the IGS-Sh-LAMP can be used as a fully instrument-free isothermal amplification device with the potential for point-of-care diagnosis in low-resource settings, especially for the detection of moderate to high *S. haematobium* infection intensities.

## Data availability statement

The original contributions presented in the study are included in the article/**Supplementary Material**, further inquiries can be directed to the corresponding author.

## Ethics statement

The studies involving humans were approved by ethical clearance was granted by the Committee of Ethics at the Ministry of Health in Madagascar (number 031-CE/MINSAN 4 June 2010). The studies were conducted in accordance with the local legislation and institutional requirements. The human samples used in this study were acquired from gifted from another research group. Written informed consent for participation was not required from the participants or the participants’ legal guardians/next of kin in accordance with the national legislation and institutional requirements.

## Author contributions

KB: Formal analysis, Investigation, Methodology, Writing – original draft, Writing – review & editing, Data curation, Visualization. EB: Conceptualization, Investigation, Methodology, Supervision, Validation, Writing – review & editing. BR: Resources, Writing – review & editing. CR: Resources, Writing – review & editing. PL: Resources, Writing – review & editing. EK: Resources, Writing – review & editing. AD: Resources, Writing – review & editing. FD: Investigation, Writing – review & editing. VS: Conceptualization, Investigation, Methodology, Supervision, Writing – review & editing. AV: Conceptualization, Investigation, Methodology, Supervision, Writing – review & editing. LL: Conceptualization, Formal analysis, Funding acquisition, Investigation, Methodology, Project administration, Supervision, Writing – original draft, Writing – review & editing.
