# Supplementary figures and images for: Next step towards point-of-care molecular diagnosis of female genital schistosomiasis (FGS): evaluation of an instrument-free LAMP procedure
Source: Front Parasitol. 2024 May 13;3:1297310. doi: 10.3389/fpara.2024.1297310 (PMC11731957; doi:10.3389/fpara.2024.1297310)

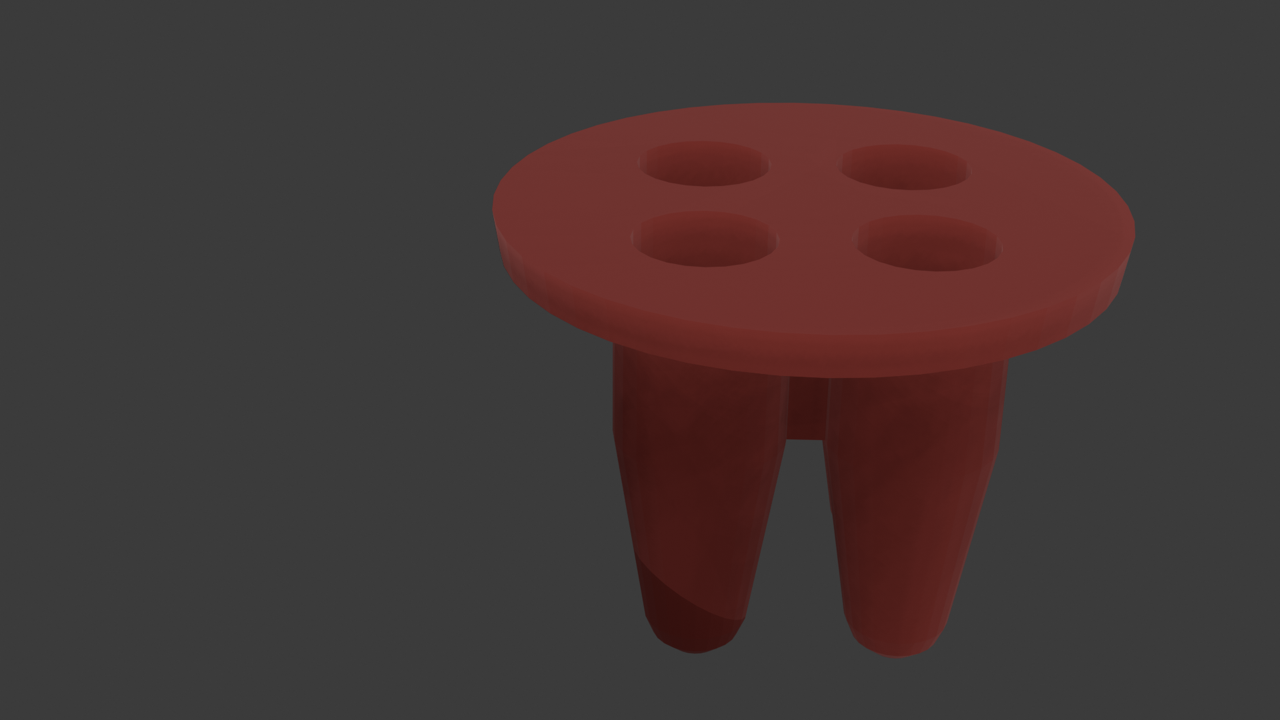

Supplement: Supplementary file 1 [file Image_1.png]
